# Hybrid Binders Through Alkaline Activation of Fine Construction and Demolition Waste

**DOI:** 10.3390/ma18143227

**Published:** 2025-07-08

**Authors:** Manuel Retamal-Rojas, Diego Aponte, William Valencia-Saavedra, Rafael Robayo-Salazar, Marilda Barra-Bizinotto

**Affiliations:** 1 Department of Civil and Environmental Engineering, Universitat Politècnica de Catalunya (UPC-BarcelonaTech), Jordi Girona 1-3, 08034 Barcelona, Spain; diego.fernando.aponte@upc.edu (D.A.); marilda.barra@upc.edu (M.B.-B.); 2 Composite Materials Group (CENM), School of Materials Engineering, Universidad del Valle, Cali 760032, Colombia; william.gustavo.valencia@correounivalle.edu.co (W.V.-S.); rafael.robayo@correounivalle.edu.co (R.R.-S.)

**Keywords:** construction and demolition waste, alkali activation, geopolymer

## Abstract

The use of construction and demolition waste (CDW) as an alternative binder to ordinary Portland cement presents a promising solution through alkaline activation. This study evaluates the physical, mechanical, and microstructural behaviour of pastes and mortars produced with CDW—specifically concrete (RH) and ceramic (RC) waste—activated with NaOH and Na_2_SiO_3_ (SS) solutions. Mortars were prepared with NaOH/SS ratios of 0.2 and 0.3 and an activator-to-precursor (AA/P) ratio of 0.2. Results showed that higher NaOH content accelerated alkaline activation, reducing setting times from 6.2 h to 3.7 h for RC and from 4.6 h to 3.2 h for RH. Conversely, increasing Na_2_SiO_3_ content led to greater drying shrinkage, from −0.42% to −0.49% in RC and from −0.46% to −0.52% in RH. Compressive strength values at 28 days ranged from 7.6 to 8.2 MPa. X-ray diffraction (XRD) revealed the presence of non-reactive crystalline phases in both precursors, while Fourier transform infrared (FTIR) spectroscopy indicated the formation of CASH, CSH, and/or (N)CASH gels. This study highlights the potential of CDW as a sustainable alternative binder and the usefulness of the proposed method for optimising alkali-activated systems, contributing to circular economy strategies in the construction sector.

## 1. Introduction

The link between climate change and human activity is unquestionable, as supported by the Global Change Research Program of the United States [1]. Observations have shown clear climate warming over the past five decades, which is primarily attributed to human emissions of greenhouse gases [2].

The cement industry plays a vital role in the global economy as it is used in the construction of various infrastructure projects. However, it is also one of the largest sources of carbon dioxide (CO_2_) emissions, contributing to over 7% of the world’s total greenhouse gas budget. The cement production process releases large amounts of CO_2_, primarily owing to the heating of raw materials such as limestone and clay, as well as inefficient production technologies. This significantly impacts the environment and contributes to global climate change [3,4,5]. Despite these challenges, the cement industry continues to grow, driven by the demand for infrastructure development in emerging economies and urbanisation in developed countries. Global cement production is estimated to reach 8.2 billion tons by 2030 [6,7].

Population growth, urbanisation, and economic development have driven the construction industry, resulting in considerable construction and demolition waste (CDW). Approximately 850 million tons of construction and demolition waste (CDW) is generated in the EU each year, accounting for 31% of the total waste generation [8].

The use of CDW as an aggregate in the preparation of new concrete has immense potential and has been a subject of research for a long time. Utilizing CDW as aggregate can reduce the use of natural aggregates and alleviate the issues associated with their extraction [9,10,11].

Currently, CDW is primarily used as a material for bulk-fill and granular layers. The coarse fraction of CDW has been extensively researched for the production of both structural and nonstructural concrete, and the use of smaller aggregates (sand) has received less attention because of its negative impact on the flowability and mechanical properties of concrete. These issues are associated with higher water absorption (recycled aggregate) compared to natural aggregates [12,13]. Among the potential materials obtained from CDW, the fine fraction is the least studied and currently the least valued.

In the quest for more environmentally friendly binding materials, new materials with lower carbon footprints have been developed through alkali activation [14,15], allowing the use of different aluminosilicates as raw materials. Alkali activation involves the chemical reaction between a solid aluminosilicate material (precursor) and an alkali activator, resulting in a product with the ability to harden [16]. Alkali activators are soluble substances that increase the pH of the reaction mixture and accelerate the dissolution of solid precursors, including hydroxides, silicates, carbonates, sulphates, aluminates, and alkali oxides [16].

The alkali activation of silica- and alumina-rich matrices, such as CDW, involves a sequence of dissolution reactions governed by alkali ion transport in a reactive porous medium. Initially, the activator (NaOH, Na_2_SiO_3_) raises the pH of the solution, promoting the dissolution of amorphous or poorly crystalline phases rich in Si and Al. This releases soluble species (e.g., Si(OH)_4_ and Al(OH)_4_^−^) [17] that subsequently polymerise to form a gel network: N-A-S-H in low-calcium systems or C-A-S-H in calcium-rich systems. The formation and structural evolution of these gels depend on both reaction kinetics and ion mobility within the matrix, closely linked to porosity development, concentration gradients, and species redistribution during drying [18,19]. The alkali activation of silica- and alumina-rich matrices, such as CDW, involves a sequence of dissolution reactions governed by alkali ion transport within a reactive porous medium. The alkali activation process of aluminosilicate materials typically proceeds through four successive stages: dissolution, depolymerisation, polymerisation, and condensation [20]. In general, the reactivity of a silico-aluminate material used as a precursor depends on various factors such as classification, origin, base material, cleanliness, and so on. As a result, the literature lacks a standardised methodology for determining the optimal amount of alkali activator in the system or the appropriate ratio between activators when more than one is used.

Some authors prefer to use the silica modulus (Ms = SiO_2_/Na_2_O) and the sodium content in the system (expressed as a percentage). For example, Gado et al. (2020) [21] investigated the alkaline activation of fired clay brick waste (grog) under different Ms values and reported maximum compressive strength at Ms = 1.25. Similarly, García-Díaz et al. (2023) [22] used ceramic waste mixed with chamotte and observed an increase in mechanical strength as Ms increased from 0.5 to 1, followed by a decrease as Ms rose from 1 to 2. Robayo-Salazar et al. (2022) [23] employed a NaOH/Na_2_SiO_3_ weight ratio of 0.25 (equivalent to Ms = 1) to activate a controlled mix of ceramic, concrete, and brick waste.

Other researchers choose to work with parameters based on the molar concentration of NaOH, while some prefer to use weight ratios between the activator and the precursor, or among the activators themselves.

The fine fraction of CDW is currently used as a filler in cementitious materials or sub-bases, which is inefficient and has a low added value [24]. Duan et al. (2020) [13] investigated the incorporation of the fine fraction of CDW as a partial replacement for OPC, yielding positive results for replacements below 30%. Robayo et al. (2022) [25] characterised alkali-activated hybrid cements using powder from concrete, ceramic, and masonry waste obtained from CDW crushing, achieving compressive strengths of up to 25.2 MPa. Borrachero et al. (2022) [24] manufactured mortars based on alkali-activated CDW using NaOH and sodium silicate as activators, achieving a compressive strength of up to 18 MPa after three days of curing at 65 °C.

In the grinding process of CDW, approximately 20% of the material generated is smaller than 150 µm [26,27]. However, this fine fraction has been underutilised in previous studies owing to its high impurity content and difficulty in proportion control. Most previous studies have attempted to crush coarse fractions rather than using naturally generated fine fractions. This is because the coarse fraction allows for greater control over the homogeneity of the composite material, facilitating the management of proportions of concrete, ceramic, and other waste materials. The precursors derived from the selection of coarse materials and their subsequent grinding may not be representative of the actual composition of construction and demolition waste (CDW). When selecting the coarse fraction, additions of ceramic, mortar, concrete, and plaster, among others, which are always present in the reject (0/5 mm) generated during the production of recycled aggregates, are not considered.

Regarding the particle size of the precursor, Bassani et al. [28] and Tan et al. [29] suggest that CDW precursors should have a particle size of less than 125 µm to achieve ideal reactivity in an alkaline medium, while Vafaei et al. (2019) [30] use a particle size of less than 70 µm for the alkali activation of ceramic waste. In this study, the fine fraction generated during the production of recycled aggregates is used, which has been ground to achieve an average particle size comparable to that of Portland cement.

The aim of this study is to assess the potential use of the fine fraction obtained from the valorisation process of CDW as recycled aggregates in the production of alkali-activated alternative binders, following a practical and logical methodology that allows for optimal dosages (by weight), both in the activator design and the activator-precursor ratio. This approach is crucial, as it directly addresses the underutilisation of this fine fraction and seeks to find a viable application for it, which could have significant implications for the sustainability and efficiency of CDW recycling.

In this study, CDW, specifically ceramic and concrete waste, is used to generate alkali-activated hybrid cements independently. Sodium hydroxide (NaOH) and sodium silicate (Na_2_SiO_3_) are used as alkali activators. Optimal dosages are determined for both precursors by varying the amount of activator in the system (by weight) as well as the NaOH/Na_2_SiO_3_ ratio (by weight). At the optimal dosages, specimens of pastes and mortars are fabricated and subjected to various tests and techniques to determine the physical, mechanical, and volumetric stability, as well as microstructural changes.

## 2. Materials and Methodology

### 2.1. Characterization of Materials

The materials used as precursors in this study were type I 52.5R (OPC) cement, in accordance with the UNE-EN 197-1 [31] standard, and construction and demolition waste (CDW), specifically ceramic waste (RC) and concrete waste (RH). The chemical compositions of these precursors were determined by X-ray fluorescence (XRF), using a Philips spectrometer, model PW2400 (Philips Analytical, Almelo, The Netherlands). The results are presented in Table 1.

The concrete waste (RH) and ceramic waste (RC) both exhibited similar chemical compositions, containing high levels of silicon (Si) and calcium (Ca). The aluminosilicate nature of the geopolymer precursors was evident, with a combined SiO_2_ + Al_2_O_3_ content of 54.2% for RH and 58.1% for RC, and SiO_2_/Al_2_O_3_ molar ratios of 13.0 and 11.4, respectively.

The determination of the density of the precursors was carried out in accordance with the UNE 80103:2013 [33] standard, while for the sand, the UNE-EN 1097-6 [34] standard was followed, including the assessment of water absorption. The densities obtained (g/cm^3^) were 2.64 for RH, 2.62 for RC, 3.11 for OPC, and 2.68 for sand. For the latter, the dry density was 2.63 g/cm^3^, with a water absorption of 0.77%.

**Figure 1:**
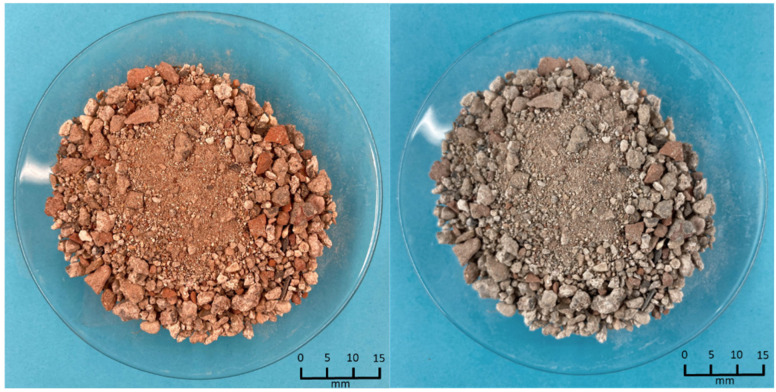
Granulometric distribution of RC (**left**) and RH (**right**).

**Figure 2:**
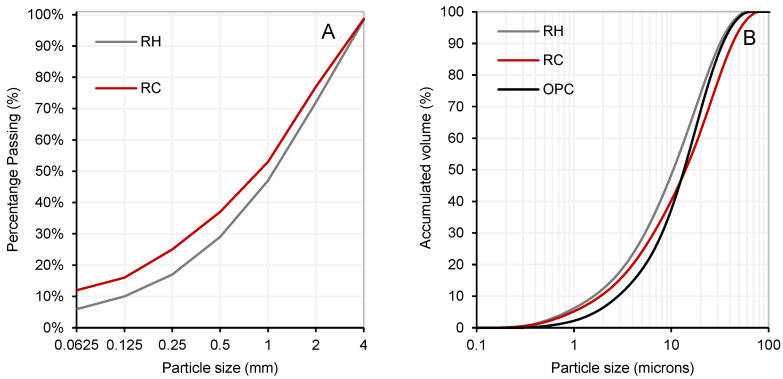
Particle size distribution before grinding (**A**) and particle size distribution of RH and RC after grinding compared to OPC (**B**).

The precursor material, obtained as a by-product of the CDW valorisation process, is shown in Figure 1. As presented in Figure 2A, the initial average particle sizes were 1.34 mm for RC and 1.57 mm for RH. Subsequently, the materials were ground to achieve a particle size distribution comparable to that of Portland cement. Figure 2B displays the particle size distribution after grinding, with average values of 14 µm for RC, 10.6 µm for RH, and 13 µm for OPC. These distributions were determined by laser granulometry (Mastersizer 2000, Malvern Instruments, Malvern, UK).

**Figure 3:**
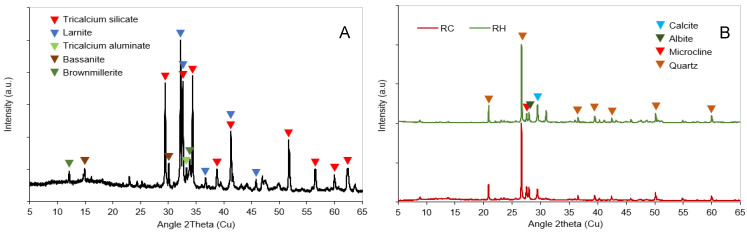
Diffractograms of precursors OPC (**A**) and CDW (**B**).

In Figure 3A, the mineralogical phases (Bruker D8-A25 diffractometer) of OPC are depicted, notably highlighting calcium silicates (tricalcium silicate and Larnite), with a smaller presence of calcium aluminates (tricalcium aluminate and brownmillerite) and gypsum (bassanite).

Both the precursors (RH and RC) exhibited similar mineralogical compositions. In Figure 3B, nearly identical crystalline phases are observed, with quartz being the main crystalline phase. Other phases were also identified, including calcite (CaCO_3_), albite (NaAlSi_3_O_8_), and microcline (KAlSi_3_O_8_).

As alkaline activators, mixtures of sodium hydroxide (NaOH) and sodium silicate (SS) or “waterglass” (Na_2_SiO_3_: SiO_2_ = 26.4%, Na_2_O = 8.0%, H_2_O = 65.6%), both commercially available, were employed.

Fourier transform infrared spectroscopy (Perkin Elmer Spectrum Two spectrometer, PerkinElmer Inc., Hopkinton, MA, USA) was used to determine the functional groups of the precursors and OPC.

**Figure 4:**
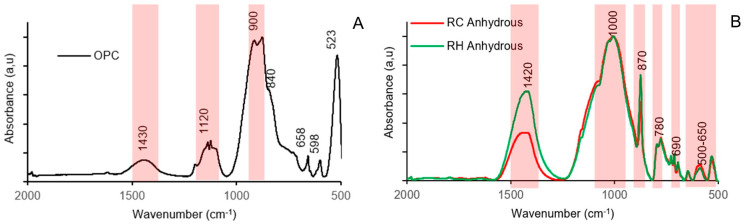
FTIR test results OPC (**A**) and CDW (**B**).

Figure 4A shows the spectrum and the main bands detected in ordinary Portland cement. Tricalcium silicate (C_3_S) was observed through strong symmetric and asymmetric stretching vibrations originating from the Si-O bonds in the SiO_4_^4−^ tetrahedral units in the 900 cm^−1^ range, as well as in the symmetric bending vibrations of the O-Si-O bond at the 523 cm^−1^ peak. Dicalcium silicate (C_2_S) was identified at the 840 cm^−1^ peak, although other absorption bands overlapped with those of C_3_S. Sulphate stretching vibrations were observed at the 1120 cm^−1^ band, which could be associated with the partial dehydration of gypsum. The asymmetric bending vibrations of the SO_4_^2−^ tetrahedra were identified at the 658 cm^−1^ and 598 cm^−1^ peaks, corresponding to bassanite and gypsum, respectively. The band at approximately 1430 cm^−1^ corresponds to the stretching vibrations in the CO_3_^2−^ groups, suggesting partial carbonation of the cement [35].

Figure 4B displays the spectra and the main bands detected in the RH and RC precursors. The bands centred at 1420, 870, and 690 cm^−1^ suggest the presence of O-C-O bonds of CO_3_^2−^ groups associated with carbonate phases owing to the presence of calcite in the precursor [26]. The main band centred at 1000 cm^−1^ corresponds to the asymmetric stretching vibrations of the Si-O-Si and Si-O-Al bonds typical of aluminosilicates. The band at 780 cm^−1^, as well as the band between 500–650 cm^−1^, is attributed to the Si-O-Si inter tetrahedral bonds in SiO_2_, suggesting the presence of quartz [36].

### 2.2. Techniques

#### 2.2.1. Pastes

The workability of the initial pastes was evaluated via visual inspection. Dosages that could not be moulded owing to rapid hardening and those that did not harden within 7 days were discarded.

Compressive strength tests on the initial pastes (phase 1) were carried out on 35 × 35 × 35 mm cubic specimens in accordance with the UNE-EN 196-1 [37] standard. A Zwick/Roell press (ZwickRolle GmbH & Co., Ulm, Germany) was used.

The fluidity of the pastes over time (Phase 2) was measured using the “Grout Spread Method”, as described in standard UNE-EN 445 [38]. Initially, the solids and liquids forming the paste were mixed for 3 min, after which the first flow measurement was performed. Subsequently, the same material was reused to measure the flow at 6, 15, and 30 min, with constant mixing for 30 s before each test.

The compressive strength test of the selected pastes (Phase 2) was performed in the same manner as previously mentioned for the pastes from Phase 1.

The determination of the initial and final setting times of the optimised pastes (Phase 3) was carried out according to the methodology described in standard UNE-EN 196-3:2017 [39].

The temperature increments in the optimised pastes (Phase 3) were measured using semi-adiabatic equipment. The solids and liquids forming the paste were mixed for only 2 min to avoid loss of information from the initial reactions. The temperature increase generated by the exothermic process of the reactions was measured using thermocouples inserted into the pastes, which transmitted the temperature changes to a data logger.

For microstructural analysis, the hydration of the pastes was stopped using the solvent exchange method [36]. The samples were pulverised to a particle size of <0.063 mm. X-ray diffraction measurements of the raw materials and optimised pastes (Phase 3) were carried out using a Bruker D8-A25 (Bruker, Billerica, MA, USA) diffractometer equipped with a Cu X-ray source (CuKα radiation, 40 kV and 40 mA) and a LynxEye position-sensitive detector (Bruker, Billerica, MA, USA). Scanning was performed between 4° and 80° in 2θ with a step size of 0.019° and a counting time of 0.8 s per step. Phase identification was carried out using the DIFFRAC EVA V5.2 software.

Fourier transform infrared spectroscopy (FTIR) was used to determine the functional groups of the precursors, ordinary Portland cement (OPC), and optimised pastes (Phase 3). A Perkin Elmer Spectrum Two spectrometer was used in the range of 4000–400 cm^−1^. Each measurement consisted of 32 scans, with a resolution of 4 cm^−1^.

#### 2.2.2. Mortars

The consistency of the mortars produced with the optimised paste dosages (Phase 3) was determined according to the methodology of the UNE-EN 1015-3 [40] standard (by flow table).

The initial shrinkage of the fresh mortars (Phase 3) was measured using an electronic device composed of six linear displacement transducers connected to a computer. Immediately after the mortar was made, it was poured into a cylindrical mould with a diameter of 100 mm and a height of 10 cm. A circular metal plate was placed inside the cylinder and on the mortar and connected to the transducers.

The density, absorption, and porosity of the hardened mortars (Phase 3) were determined according to the methodology of standard ASTM C 642-13 [41].

Shrinkage by drying in the mortar (Phase 3) was measured as described in the ASTM C490-04 [42] standard. Specimens of 25 × 25 × 250 millimetres were fabricated according to standard UNE-EN 196-1:2018 [37]. For each type of mortar, four specimens were prepared, of which two were kept in a humid chamber (95% humidity and 21 °C) and 2 in a room at ambient temperature (60% humidity and 21 °C). A Sylvac S229 electronic micrometre (Sylvac SA, Le Landeron, Switzerland) was used for the measurements.

To record the mass change caused by drying in the mortars (Phase 3), the same specimens used in the drying shrinkage test were weighed on a digital scale.

### 2.3. Experimental Methodology

**Figure 5:**
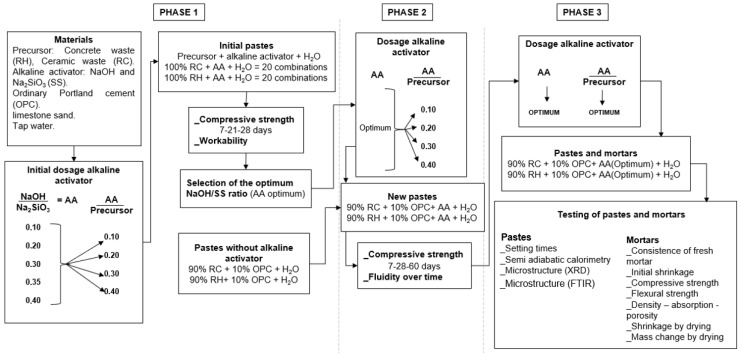
Flowchart of materials and methods.

The research methodology was conducted according to the scheme illustrated in Figure 5. This study was divided into three phases.

Phase 1:

Once the materials were characterised, the initial doses of the alkaline activator were prepared. The NaOH/SS ratios (AA) were 10%, 20%, 30%, 35%, and 40% (by weight), whereas the activator-to-precursor ratios (AA/precursor) were 10%, 20%, 30%, and 40% (by weight). The liquid-to-solid ratio (L/S) was 0.4 for all samples, where “L” represents the sum of mixing water plus the water contributed by Na_2_SiO_3_ and “S” represents the sum of solids, including the precursor, NaOH, and the percentage of Na_2_SiO_3_ that is not water (by weight).

Using the dosages of the alkaline activator, 20 initial pastes of RC and RH were prepared. Cubic specimens measuring 35 × 35 × 35 mm were manufactured. The specimens were tested for compression at 7, 21, and 28 days. All the specimens were kept in a humid chamber (95% humidity and 21 °C) from the day of fabrication until the testing age.

Based on the compression strength and workability results, the NaOH/SS ratio with the best performance was selected (optimal NaOH).

Cubic specimens were also manufactured without an alkaline activator, with 10% replacement of OPC compared to the precursor. The specimens were subjected to compression testing for 7, 28, and 60 days.

Phase 2:

With the optimal dosages of NaOH/SS ratio (AA), new pastes were prepared while maintaining the AA/precursor ratio at 10%, 20%, 30%, and 40%. For these new pastes, 10% (by weight) of the precursor was replaced by OPC. The liquid-to-solid ratio (L/S) was set at 0.4 for all samples. Flow tests were conducted over time, and compression strength tests were performed at 7, 28, and 60 days using the new pastes.

Phase 3:

Based on the results of the Phase 2 tests, the optimal AA/precursor ratios were identified. Using the optimal NaOH/SS (optimal AA) and AA/precursor ratios, pastes and mortars were prepared with precursors comprising 90% RH + 10% OPC and 90% RC + 10% OPC (by weight). The optimal pastes were subjected to setting time tests, semi-adiabatic calorimetry, and microstructural analysis (XRD).

In the case of mortars, dosages were made by weight, maintaining a sand-to-precursor (CDW + OPC) ratio of 3.2 to 1 (3.2:1). The liquid-to-solid ratio (L/S) was set at 0.4 for all samples. The mortar specimens were demolded and placed in a humidity chamber 24 h after fabrication, where they remained until the testing age. These mortars were subjected to assessments of fresh mortar consistency, initial shrinkage, compressive strength, flexural strength, density–absorption–porosity, shrinkage by drying, and mass change by drying.

## 3. Results and Discussion

### 3.1. Initial Dosages to Determine Optimum NaOH/Na_2_SiO_3_ Ratio

Table 2 lists the composition of the initial pastes (0% OPC). The pastes were manufactured using RH and RC as precursors (P). The NaOH/Na_2_SiO_3_ ratio (AA) and L/S ratio are as indicated in Phase 1. For each combination, a series of cubic specimens (35 × 35 × 35 mm) were fabricated and subjected to compression tests at ages of 7, 21, and 28 days.

**Figure 6:**
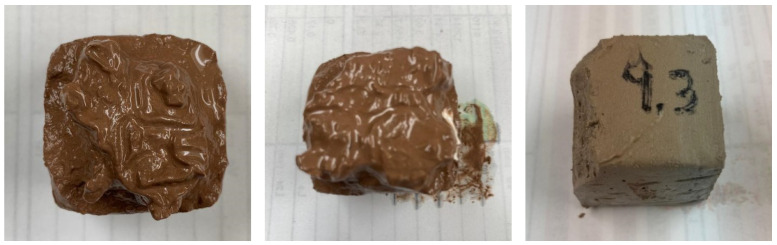
Unhardened paste or paste without compressive strength.

**Figure 7:**
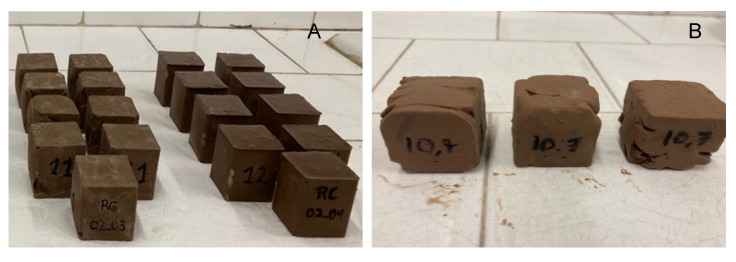
Tested hardened specimens (**A**) and hardened paste before moulding (**B**).

Pastes with a low NaOH/Na_2_SiO_3_ ratio (AA = 10%) either failed to harden or did not attain sufficient strength to undergo compression testing (Figure 6). Converely, pastes with a high NaOH/Na_2_SiO_3_ ratio (AA = 40%) hardened rapidly, even before being moulded (Figure 7B).

**Figure 8:**
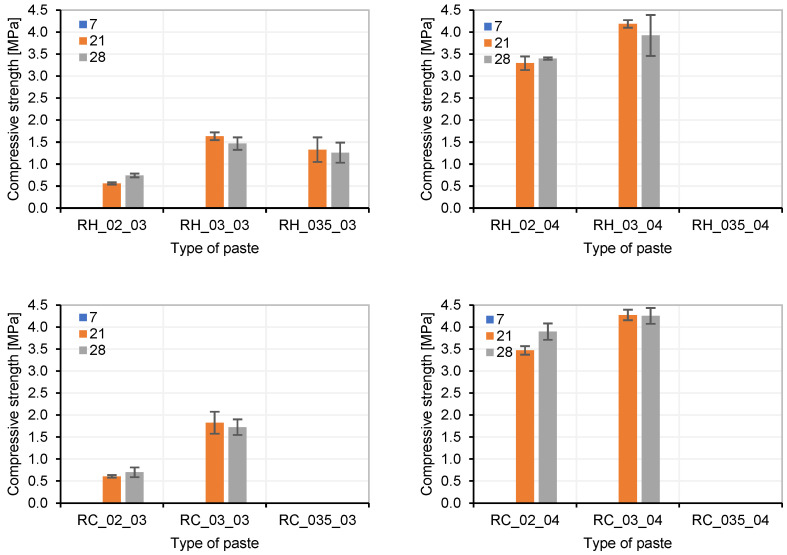
Compression strength of RH and RC pastes with different AA and AA/P ratios.

Finally, the compressive strength results of the pastes that were in conditions to be tested (Figure 7A) are displayed in Figure 8. It was not possible to conduct tests on the pastes at the 7-day age because they had not hardened or developed sufficient strength.

For both precursors (RH and RC), similar behaviour was observed in the pastes. Dosages with NaOH/Na_2_SiO_3_ ratios of 20% and 30% exhibit better performance in terms of workability and compressive strength. These percentages were considered to be optimal for NaOH/Na_2_SiO_3_ (Optimal AA = 20% and 30%).

**Figure 9:**
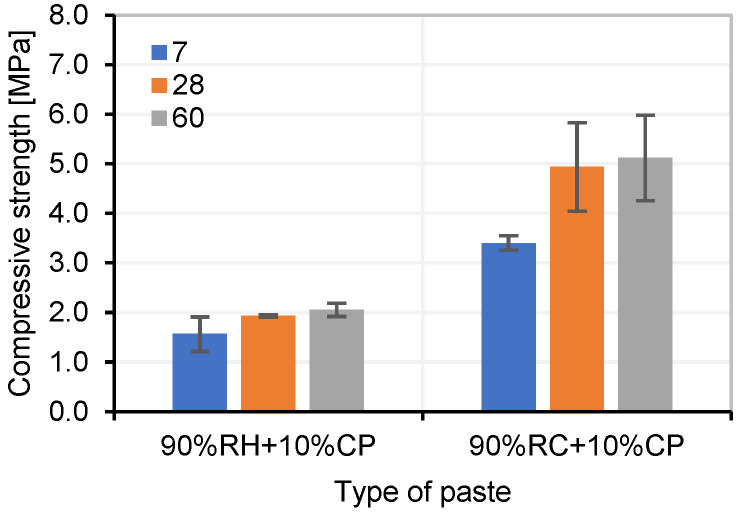
Compression strength of RH and RC pastes without alkaline activator (10% OPC).

Cubes were also produced with pastes without an alkaline activator by substituting 10% of the precursor with OPC and maintaining the liquid-to-solid ratio (L/S = 0.4). These samples demonstrated that by adding 10% OPC (as a replacement for the precursor), the pastes hardened and could be demoulded within 24 h. The specimens were subjected to compressive strength testing, and the results are shown in Figure 9.

### 3.2. Dosages to Determine Optimum Alkaline Activator/Precursor Ratio (AA/0.9P + 0.1OPC)

Based on the optimal NaOH/Na_2_SiO_3_ ratios (Optimal AA = 20% and 30%) determined in Section 3.1, pastes were manufactured by varying the activator/precursor ratio AA/(0.9P + 0.1OPC): 10%, 20%, 30%, and 40%. The combinations obtained are listed in Table 3. The liquid/solid ratio was kept constant for all samples (L/S: 0.4)

**Figure 10:**
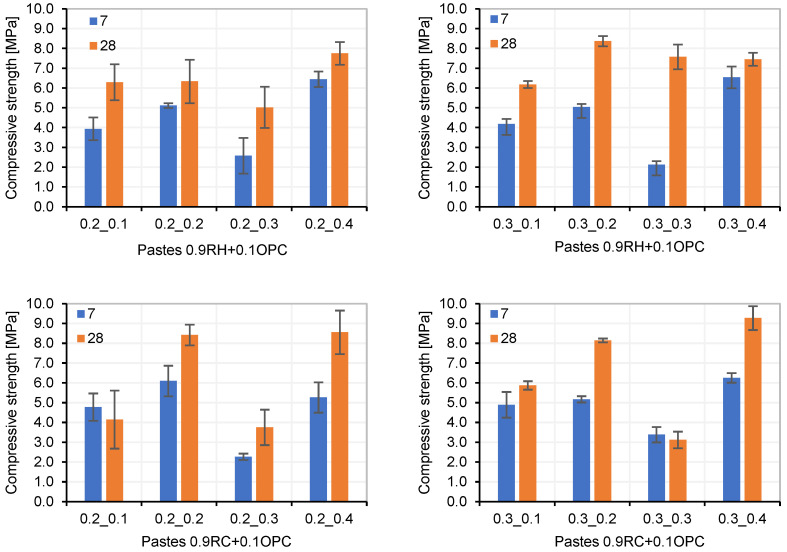
Compression strength of RH and RC pastes with optimal AA ratio and different AA/(0.9P + 0.1OPC) ratios.

**Figure 11:**
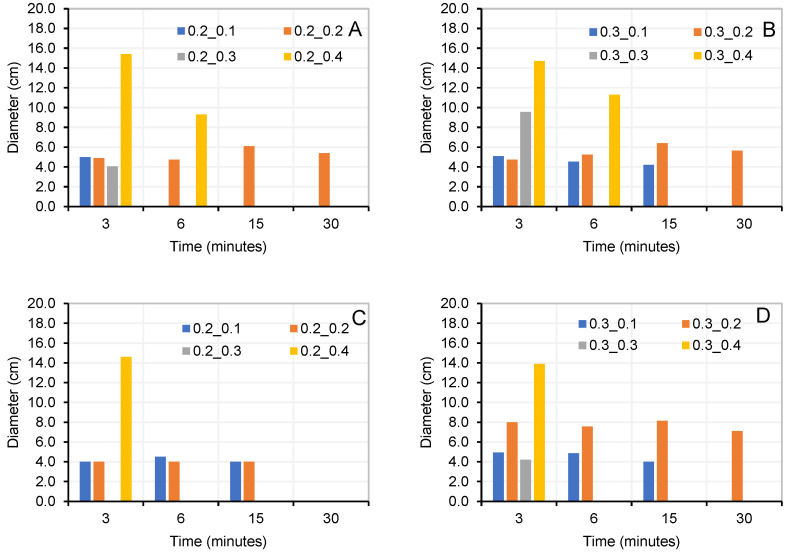
Loss of workability over time of RH and RC pastes with optimal AA ratios and different AA/P ratios (10% OPC by weight): (**A**,**B**) (0.9RH + 0.1OPC); (**C**,**D**) (0.9RC + 0.1OPC).

The manufactured pastes were tested for compression at ages of 7 and 28 d (Figure 10). Workability loss over time was measured by conducting flow tests at 3, 6, 15, and 30 min (Figure 11).

Pastes with an activator/precursor ratio of 20% (AA/(0.9P + 0.1OPC): 20%) maintained their workability for a longer duration for both precursors. This condition was prioritised when selecting the optimal AA/(0.9P + 0.1OPC) ratio. Other dosages achieved higher mechanical strengths but with very low workability times. An example of this is seen in the dosages 0.9RH + 0.1OPC_02_04 and 0.9RC + 0.1OPC-03-04 (Figure 10), both with higher mechanical strengths than the optimal combination, but with workability times of 6 and 3 min, respectively (Figure 11A and Figure 11D, respectively).

The flowability of the pastes (Figure 11) was measured until it was no longer possible to mould them in the cylinder. In Figure 11, pastes that maintain diameters close to 40 mm over time (for example, Figure 11C, paste 0.2-0.2) indicate that the paste did not flow but had not yet hardened.

**Figure 12:**
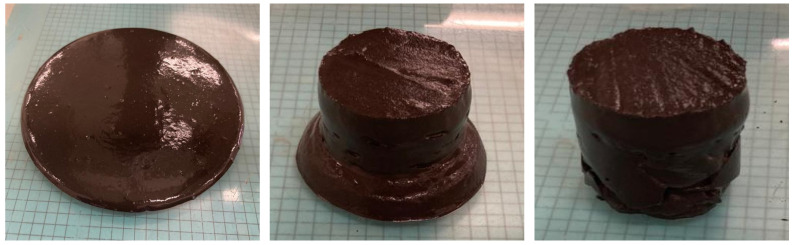
Loss of workability over time.

Figure 12 shows the sequence of workability loss over time for one of the analysed pastes.

### 3.3. Optimised Dosages for Pastes and Mortars

Finally, based on the tests conducted in Section 3.1 and Section 3.2, the optimal dosages were determined and are presented in Table 4. Using these dosages, pastes and mortars were manufactured for both precursors and their physical and mechanical properties, as well as mineralogical changes at different ages, were analysed.

### 3.4. Testing of Pastes and Mortars

**Figure 13:**
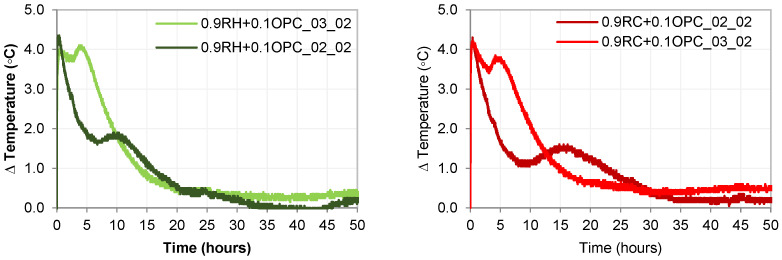
Semi-adiabatic calorimetry (RH and RC pastes).

As observed in Figure 13, the normalised heat flow curves were similar for all four pastes. All the pastes exhibited two main peaks, albeit at different times. This similarity in the heat flow profile indicates a resemblance in the reaction mechanism between the alkali-activated precursors RH and RC systems.

The calorimetric curves obtained for samples 0.9RH + 0.1OPC_02_02, 0.9RH + 0.1OPC_03_02, 0.9RC + 0.1OPC_02_02, and 0.9RC + 0.1OPC_03_02 revealed the main stages of reactions typically occurring in the ordinary Portland cement hydration process [43]. These stages are commonly known as the initial wetting, induction, rapid acceleration, deceleration, and steady-state periods [44,45]. The primary difference between the samples containing 0.9P + 0.1OPC-02-02 and 0.9P + 0.1OPC-03-02 (P: Precursor RH and RC) appears to be that the presence of a higher NaOH/Na_2_SiO_3_ ratio (higher Na content in the system) accelerates the reaction, providing a very short induction period and a higher peak during the main reaction (second peak). For 0.9P + 0.1OPC_03_02, it can be easily observed in the hydration curve that the main reaction (second peak) and initial wetting (first peak) almost overlap. A higher percentage of NaOH in the system increases the spontaneity of the reaction, leading to a combined major peak. This condition allows the formation of hydration products during the early hours of the hydration process.

**Figure 14:**
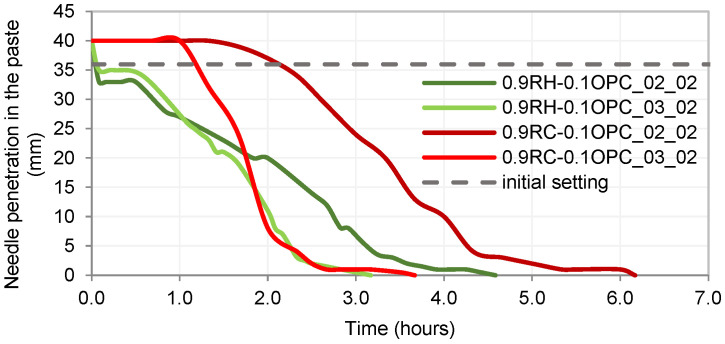
Setting times for RH and RC pastes.

Figure 14 shows the setting time curves for the four examined pastes. It is evident that the 0.9P + 0.1OPC_03_02 pastes exhibited a faster setting time compared to the 0.9P + 0.1OPC_02_02 pastes, even though the initial setting time was nearly the same for the RH precursor pastes. This aligns with the calorimetry results (Figure 13), revealing an acceleration of the reactions in pastes with a higher concentration of NaOH (0.9P + 0.1OPC_03_02).

The pastes 0.9RH + 0.1OPC_02_02 and 0.9RH + 0.1OPC_02_03 initiated setting within a few minutes, in contrast to what occurred with the RC pastes. RC pastes allow for a longer initial working time; however, once the setting begins, the curves have a steeper slope than the RH curves.

**Figure 15:**
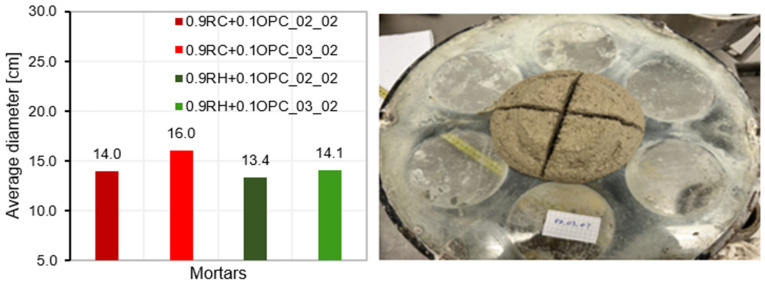
Consistency of fresh mortar.

Figure 15 shows the test results for the four mortar mixtures. All four mortars demonstrated good consistency, with 0.9RC+0.1OPC_03_02 achieving the widest spread on the flow table (16 cm), whereas 0.9RH + 0.1OPC-02-02 exhibited the lowest flow.

**Figure 16:**
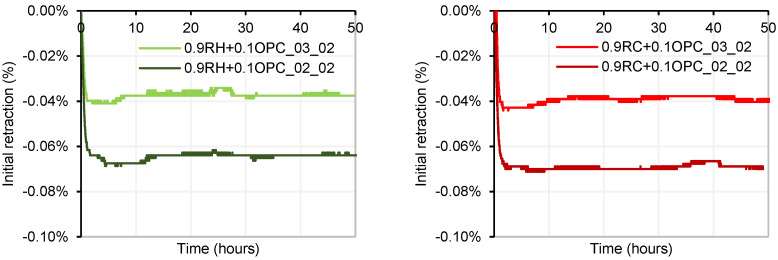
Initial shrinkage of RH mortar (**left**) and RC mortar (**right**).

Using sensors, the initial shrinkage or shrinkage in the fresh state was measured for the four manufactured mortars. In Figure 16, it can be observed that mortars with a lower NaOH/Na_2_SiO_3_ ratio (NaOH/SS: 0.2), that is, precursors activated with a greater quantity of SS in the system, exhibit greater initial shrinkage than precursors activated with a NaOH/SS ratio of 0.3 for both precursors (RH and RC). This behaviour is consistent with the results of shrinkage by drying, analysed subsequently. The reduction from 30% to 20% in the NaOH/SS ratio resulted in an increase in shrinkage of approximately 75% for both precursors.

**Figure 17:**
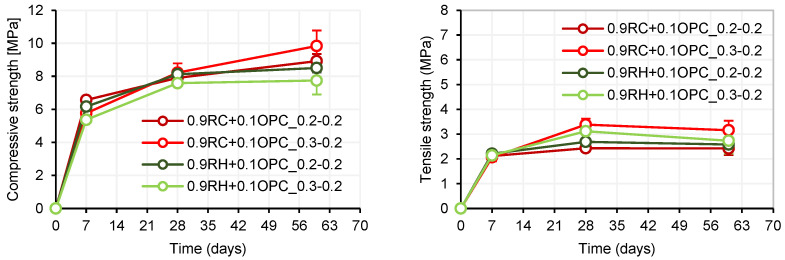
Compressive strength (**left**) and flexural strength (**right**).

The compression and flexural strength results of the mortars with alkali-activated RC and RH precursors are shown in Figure 17. Mortars with RC precursors exhibited higher compressive strengths for both dosages than those with RH precursors. In Figure 17, it can be observed that, in RC mortars, a higher NaOH/SS ratio (NaOH/SS: 0.3) yields higher mechanical strengths. However, the opposite occurs in RH mortars; the highest strength is achieved for 09RH + 0.1OPC_0.2_0.2 (NaOH/SS: 0.2). This may be due to the nature of the precursors; the RC material contains a higher amount of aluminosilicates than the RH precursor (see Table 1), implying the need for a higher NaOH concentration in the system to break the bonds of the available material during the alkaline activation process. It is possible that, for the RH precursor, a NaOH/SS ratio of 0.2 is sufficient to achieve optimal alkaline activation. Therefore, higher amounts in this ratio would not be necessary, as there is no more material available for the chemical reaction between the aluminosilicates and the alkali activator. In fact, a NaOH/SS ratio of 0.3 reduced the compressive strength because it decreased the contribution of SS in the system, thus reducing the SiO_3_ contribution as well.

**Figure 18:**
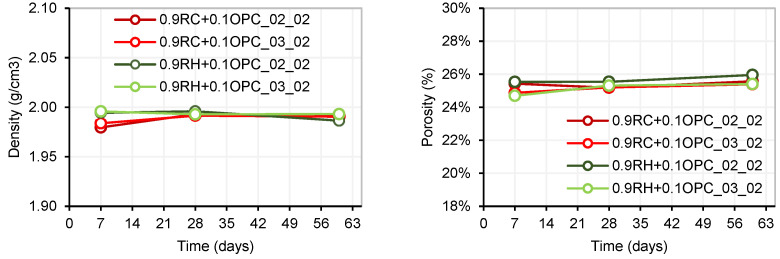
Density (**left**) and porosity (**right**).

The density and porosity of the four mortar dosages were measured at 7, 28, and 60 d. It can be observed in Figure 18 that there were practically no changes in these parameters over time, remaining relatively constant in all samples.

**Figure 19:**
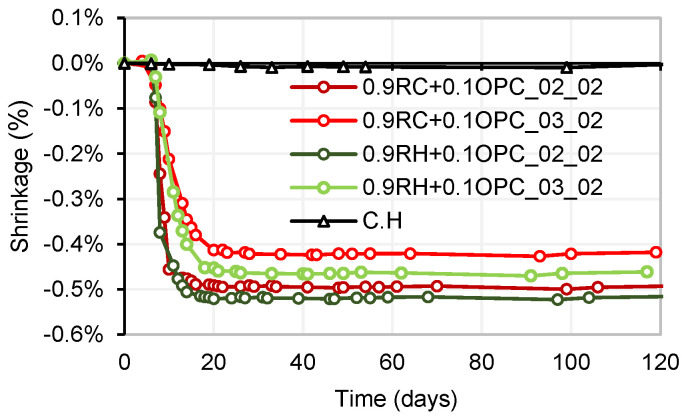
Shrinkage by drying (room temperature).

An analysis of shrinkage by drying over time was conducted on four types of manufactured mortars. Mixtures with a NaOH/Na_2_SiO_3_ ratio of 20% (referred to as 0.9P + 0.1OPC_02_02) exhibited more pronounced shrinkage compared to mixtures with a NaOH/Na_2_SiO_3_ ratio of 30% (referred to as 0.9P + 0.1OPC_03_02), for both precursors P: RH and P: RC. Figure 19 illustrates the maximum shrinkage observed for the four mortars. The highest shrinkage was recorded in mortar 0.9RH + 0.1OPC_02_02, reaching a value of −0.52%. This shrinkage remained constant from 18 days onwards. Mortars 0.9RC+0.1OPC_02_02, 0.9RH + 0.1OPC_03_02, and 0.9RC + 0.1OPC_03_02 registered shrinkages of −0.49%, −0.46%, and −0.42%, respectively, stabilizing at 16, 21, and 23 days. These findings align with those of previous studies [45,46], where it was observed that samples with higher concentrations of SiO_2_ in the activator exhibited higher levels of shrinkage by drying. In other words, an increase in the silica modulus (SiO_2_/Na_2_O) for a constant sodium concentration results in greater shrinkage compared with an increase in sodium concentration while maintaining a constant silica modulus. For each dosage, four specimens were fabricated, of which two were kept at room temperature and two in a humidity chamber (C.H).

**Figure 20:**
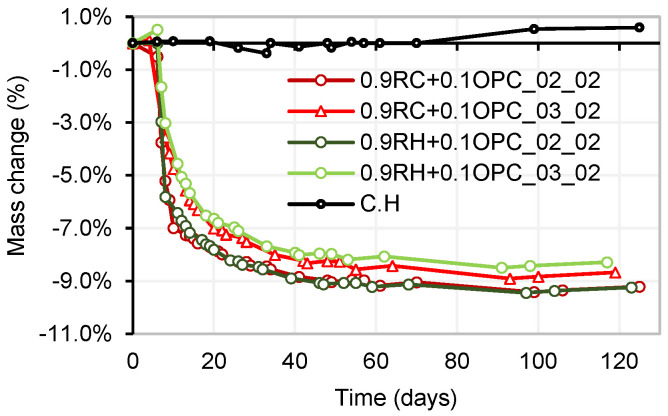
Mass change by drying (room temperature).

Parallel to the study of shrinkage by drying, the mass change due to drying was analysed in the samples. As expected, the mass change in each group of samples is directly related to the shrinkage caused by drying. The specimens with a dosage of 0.9P + 0.1OPC_02_02 (for both precursors) exhibited a greater mass loss than the 0.9P + 0.1OPC_03_02 dosage mortars. Figure 20 presents the mass losses (in percentages) over time for the four mortars.

**Figure 21:**
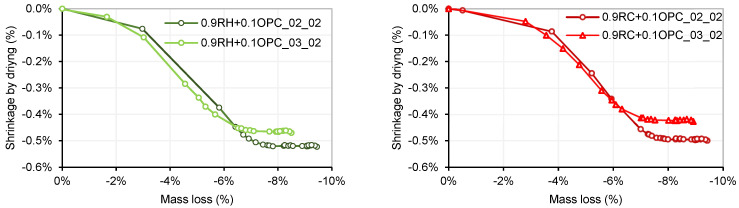
Shrinkage by drying vs. mass change by drying.

Figure 21 shows the maximum mass loss resulting from the stabilisation of the drying shrinkage in the different mortar mix designs tested. Mortar 0.9RH + 0.1OPC_02_02 experienced a maximum drying shrinkage of 0.52%, which correlated with a mass loss of 9.4%. Mortars 0.9RH + 0.1OPC_03_02, 0.9RC + 0.1OPC_02_02, and 0.9RC + 0.1OPC_03_02 exhibited maximum shrinkages of 0.46%, 0.49%, and 0.42%, respectively, associated with maximum mass losses of 8.5%, 9.4%, and 8.9%, respectively.

**Figure 22:**
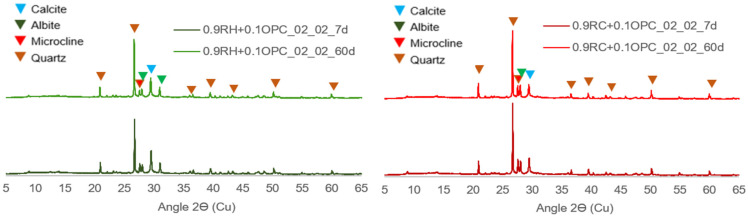
XRD at 7 and 60 d in pastes 09RH+0.1OPC_02_02 (**left**) and 09RC+0.1OPC_02_02 (**right**).

The changes in the crystalline phases of the four optimised pastes were analysed using XRD. As depicted in Figure 22, crystalline phases such as quartz, calcite, and albite (present in the anhydrous precursor) remain virtually constant in the activated pastes, even with no significant difference between the 7- and 60-day ages, indicating that these phases are nonreactive under the conditions of this study. The low reactivities of these phases have been identified by other authors in similar studies [47,48,49].

**Figure 23:**
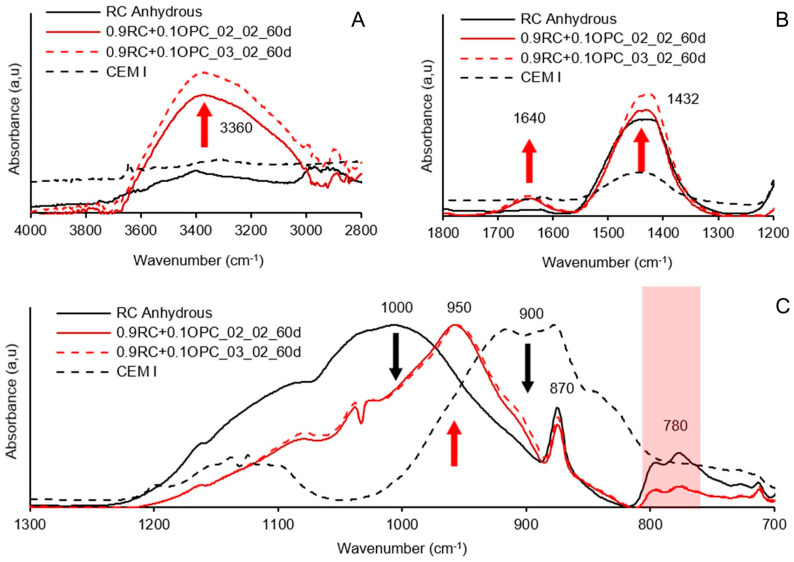
Results of FTIR tests for pastes activated 0.9RC + 0.1OPC_02_02 and 0.9RC + 0.1OPC_03_02 at 60 d of age. Wavenumber 2800–4000 (**A**), 1200–1800 (**B**) and 700–1300 (**C**).

**Figure 24:**
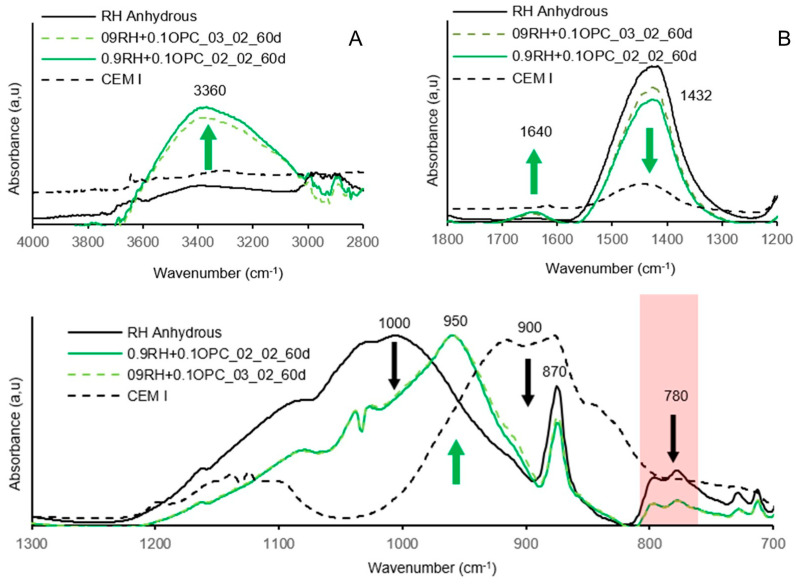
Results of FTIR tests for pastes activated 09RH + 0.1OPC_02_02 and 09RH + 0.1OPC_03_02 at 60 d of age. Wavenumber 2800–4000 (**A**), 1200–1800 (**B**) and 700–1300 (**C**).

The FTIR spectra of the alkali-activated pastes with RC and RH precursors are shown in Figure 23 and Figure 24, respectively. For comparison, the FTIR spectra of the anhydrous raw materials are also included.

The broad band centred at 3360 cm^−1^ is associated with the stretching vibrations of the O-H bond, indicating the presence of adsorbed water in alkali-activated materials [20,35]. In Figure 23A, it is observed that the curve centred at 3360 cm^−1^ is higher in the paste with the dosage of 0.9RC + 0.1OPC_03_02 compared to 0.9RC + 0.1OPC_02_02. The same phenomenon is observed in Figure 24A for pastes with the RH precursor; however, in this case, the intensity is higher in the paste 09RH+0.1OPC_02_02. The vibrations of the O-H bond in this band (3360 cm^−1^) are directly related to the formation of the gel and, hence, to the mechanical strength. The greater mechanical strength of mortars manufactured with RC and RH precursors is associated with dosages of 0.9RC + 0.1OPC_03_02 and 09RH+0.1OPC_02_02 (Figure 17), which fully coincides with the higher intensity of the curves formed in the 3360 cm^−1^ band.

In Figure 23 and Figure 24, the peaks centred at 1432 cm^−1^ and 870 cm^−1^ can be associated with the O-C-O bonds of the carbonate group (CO_3_^2−^) due to the presence of calcite in the anhydrous precursor (RC and RH) [22,49]. The peak observed around 1640 cm^−1^ is attributed to the stretching vibrations of the –OH group, as well as the bending vibrations of the H–O–H bond corresponding to retained water molecules [20]. These signals originate from water adsorbed on the surface or confined within internal cavities of the alkali-activated cement structure. In Figure 23A, an increase in the characteristic carbonate peak was observed in the alkali-activated RC pastes, suggesting an excess of Na+ that remained unreacted after the formation of Si and Al tetrahedra, ultimately resulting in carbonation. No efflorescence was visually evident in any test specimen.

In the case of the RH pastes, the carbonate peak intensity decreased. This could be due to part of the calcium composing the carbonate reacting during the alkali activation process owing to the lower amount of available Si and Al in the anhydrous precursor.

The Si-O-Si and Si-O-Al bonds present in the anhydrous precursors RC and RH are represented by the peaks centred at 1000 cm^−1^ [22,47,49] in Figure 23 and Figure 24, respectively. When a material rich in aluminosilicates comes into contact with an alkaline solution, these bonds break. The same phenomenon occurs with the Si-O bonds present in the curve of anhydrous OPC (peak 900 cm^−1^).

The precipitation of these elements and their subsequent reorganisation led to the formation of the gel, as manifested by the peak formed at 950 cm^−1^ in the curve corresponding to the alkali-activated precursor (Figure 23 and Figure 24). The significant width of the main band (950 cm^−1^) may indicate the coexistence of various types of gels, such as CASH, CSH, and hybrid (N)CASH. The prominent band centred at 950 cm^−1^ is attributed to the asymmetric stretching vibration of the Si-O-Si and Si-O-Al bonds [20].

The double peak observed for both precursors in the 780 cm^−1^ band (Figure 23C and Figure 24C) is attributed to the bending of Si-O-Si bonds in SiO_2_, [49] suggesting the presence of quartz. In both graphs, a decrease in the double peak was observed for the alkali-activated precursors, indicating weak dissolution of the quartz phases under the study conditions.

## 4. Conclusions

This study confirms the technical feasibility of using the fine fraction of construction and demolition waste (CDW), particularly ceramic (RC) and concrete (RH) waste, as precursors for alkali-activated binders. A practical dosage approach enabled the identification of optimal NaOH/Na_2_SiO_3_ and activator/precursor (AA/P) ratios, specifically 0.2 and 0.3 for NaOH/SS (with AA/P = 0.2), corresponding to silica moduli (Ms) of 0.87 and 1.16, respectively. However, the methodology shows a limitation, as 10% variations in NaOH/SS ratios cause significant shifts in Ms values.

The mortars made with RC achieved compressive strengths of 8.9 and 9.8 MPa at 60 days for NaOH/SS ratios of 0.2 and 0.3, respectively. In comparison, RH mortars reached lower values of 7.8 and 8.5 MPa under the same conditions. Setting times in RC mixtures were 6.2 h and 3.7 h for NaOH/SS ratios of 0.2 and 0.3, respectively. For RH mortars, setting times were 4.6 h and 3.2 h for ratios of 0.3 and 0.2, respectively. The greatest drying shrinkage in both RC and RH was observed with a NaOH/SS ratio of 0.2, reaching −0.49% and −0.52%, respectively. These shrinkage values were accompanied by mass losses of −9.21% for RC and −9.25% for RH.

XRD analysis confirmed the persistence of low-reactivity crystalline phases, while FTIR spectra evidenced the formation of aluminosilicate polymerisation products and hybrid gels such as CASH, CSH, and (N)CASH.

These findings reinforce the potential of CDW fine fractions as a sustainable, low-impact binder for non-structural applications, without requiring thermal or chemical pre-treatment. Further detailed analyses are needed to characterise gel evolution and binding mechanisms more precisely.

## Figures and Tables

**Table 1:** Chemical properties of the waste and Portland cement [32].

Material	SiO_2_	Al_2_O_3_	Fe_2_O_3_	CaO	MgO	K_2_O	MnO	SO_3_	Others	LOI
RH	47.93	6.25	2.31	18.52	2.82	2.06	0.04	0.50	1.22	18.35
RC	58.13	8.96	3.20	13.04	1.10	2.53	0.05	1.17	1.68	10.14
OPC	19.96	4.68	3.32	63.27	1.52	0.81	0.03	3.11	0.67	2.63

**Table 2:** Initial dosages to determine the best NaOH/ Na_2_SiO_3_ (AA) ratio based on the compressive strength.

Type of Paste	NaOH/ Na_2_SiO_3_	AA/P	L/S
P_01_01	0.1	0.1	0.4
P_01_02		0.2	
P_01_03		0.3	
P_01_04		0.4	
P_02_01	0.2	0.1	
P_02_02		0.2	
P_02_03		0.3	
P_02_04		0.4	
P_03_01	0.3	0.1	
P_03_02		0.2	
P_03_03		0.3	
P_03_04		0.4	
P_035_01		0.1	
P_035_02	0.35	0.2	
P_035_03		0.3	
P_035_04		0.4	
P_04_01	0.4	0.1	
P_04_02		0.2	
P_04_03		0.3	
P_04_04		0.4	

**Table 3:** Dosage for determining the best AA/(0.9P + 0.1OPC) ratio based on compressive strength and workability.

Type of Paste	NaOH/ Na_2_SiO_3_	AA/(0.9P + 0.1OP)	L/S
0.9P + 0.1OPC_02_01	0.2	0.1	0.4
0.9P + 0.1OPC_02_02		0.2	
0.9P + 0.1OPC_02_03		0.3	
0.9P + 0.1OPC_02_04		0.4	
0.9P + 0.1OPC_03_01	0.3	0.1	
0.9P + 0.1OPC_03_02		0.2	
0.9P + 0.1OPC_03_03		0.3	
0.9P + 0.1OPC_03_04		0.4	

**Table 4:** Optimised dosages.

Type of Paste	NaOH/ Na_2_SiO_3_	AA/(0.9P + 0.1OP)	L/S
0.9P + 0.1OPC_02_02	0.2	0.2	0.4
0.9P + 0.1OPC_03_02	0.3	0.2	

## Data Availability

The original contributions presented in this study are included in the article. Further inquiries can be directed to the corresponding author.
